# No impact of transgenic *cry1C* rice on the rove beetle *Paederus fuscipes*, a generalist predator of brown planthopper *Nilaparvata lugens*

**DOI:** 10.1038/srep30303

**Published:** 2016-07-22

**Authors:** Jiarong Meng, Juma Ibrahim Mabubu, Yu Han, Yueping He, Jing Zhao, Hongxia Hua, Yanni Feng, Gang Wu

**Affiliations:** 1 College of Plant Science and Technology, Huazhong Agricultural University, Wuhan 430070, China

## Abstract

T1C-19 is newly developed transgenic rice active against lepidopteran pests, and expresses a synthesized *cry1C* gene driven by the maize ubiquitin promoter. The brown planthopper, *Nilaparvata lugens*, is a major non-target pest of rice, and the rove beetle (*Paederus fuscipes*) is a generalist predator of *N. lugens* nymphs. As *P. fuscipes* may be exposed to the Cry1C protein through preying on *N. lugens*, it is essential to assess the potential effects of transgenic *cry1C* rice on this predator. In this study, two experiments (a direct feeding experiment and a tritrophic experiment) were conducted to evaluate the ecological risk of *cry1C* rice to *P. fuscipes*. No significant negative effects were observed in the development, survival, female ratio and body weight of *P. fuscipes* in both treatments of direct exposure to elevated doses of Cry1C protein and prey-mediated exposure to realistic doses of the protein. This indicated that *cry1C* rice had no detrimental effects on *P. fuscipes*. This work represents the first study of an assessment continuum for the effects of transgenic *cry1C* rice on *P. fuscipes*. Use of the rove beetle as an indicator species to assess potential effects of genetically modified crops on non-target arthropods is feasible.

Rice (*Oryza sativa* L.) is one of the most important staple foods in the world. More than 50% of the world population (or more than 3 billion people) depend on rice for their daily lives^1^. The annual total planting area for rice was 29.4 million hectares in China for 2006, and China produced 29% of the world’s rice^2^,^3^. However, rice in China suffers from many insect pests, including planthoppers, *Nilaparvata lugens* (Stål), *Sogatella furcifera* (Horváth) and *Laodelphax striatellus* (Fallen); rice borers, *Chilo suppressalis* (Walker) and *Tryporyza incertulas* (Walker). In some regions, the water weevil *Lissorhoptrus oryzophilus* Kuschel, the gall midge *Orseolia oryzae* (Wood-Mason), and the thrip *Chloethrips oryzae* (Wil.) also heavily infest rice^4^.

To meet demands for feeding its growing population and replacing the intense utilization of insecticides in controlling pests, China has devoted great efforts to develop insect-resistant genetically modified (GM) rice lines^5^,^6^. Although no GM rice line has yet been commercialized, many transgenic lines expressing Bt proteins against lepidopteran pests, for example those expressing *cry1Ab*, *cry1Ac*, *cry2Aa*, *cry1Fa* and *cry1Ca* have been developed, and these exhibit high resistance against lepidopteran target pestss^7^,^8^,^9^. To avoid potential risks to the environment associated with cultivating GM plants, any new GM plant needs to undergo a rigorous environmental risk assessment prior to its approval for commercial cultivation. An important part of this assessment, especially in the case of insect-resistant GM plants, is the evaluation of potential effects on valued non-target organisms^6^,^10^.

The brown planthopper (BPH), *Nilaparvata lugens*, is the important herbivorous insect of rice by sucking the phloem sap and causes huge yield loss^11^,^12^,^13^. It lives in rice paddies and is a significant aggressive predator of *N. lugens*^14^. It lives in the rice paddy and is recognized as a significant aggressive predator of *N. lugens*^15^. Since *P. fuscipes* is likely to be exposed to Cry proteins in rice fields by preying on *N. lugens*, the potential effects of transgenic Bt rice on this non-target natural predator should be evaluated case-by-case prior to the commercialization of transgenic Bt rice.

The Bt rice line T1C-19 expresses Cry1C protein and shows high resistance against rice leaf folder, *Cnaphalocrocis medinalis* Guenee^16^. Based on the risk assessment guidelines for insect-resistant GM crops, impacts of transgenic *cry1C* rice on non-target predators have already been evaluated. Laboratory studies indicated that larvae and adults of *Chrysoperla subpiraticus* (Neuroptera: Chrysopidae) are not sensitive to Cry1C proteins when provided in artificial diets or Bt rice pollen^17^. *Chrysoperla sinica* is a prevalent predator species in Chinese rice fields, its larvae may consume planthoppers and its adults feed primarily on pollen, honeydew and nectar^18^. High dosage of Cry1C proteins in the artificial diet had no detrimental impacts on the life-table parameters of *Propylaea japonica* (Coleoptera: Coccinellidae), and hence the growing of transgenic *cry1C* rice should pose a negligible risk to *P. japonica*^19^. *P. japonica* is a common and abundant predator in rice paddy, both the larvae and adults consume thrips, eggs and young larvae of Lepidoptera, and the adults also feed on rice pollen^20^,^21^. Cry1C had no significant adverse effects on the population dynamics of arthropod predators^13^,^22^. However, there are no reports concerning impacts of Cry1C on *P. fuscipes*.

In this study, a tritrophic bioassay was conducted to assess whether T1C-19 plants had prey-mediated effects on the life-table parameters of *P. fuscipes*, when *P. fuscipes* preyed on *N. lugens* nymphs fed on T1C-19. The biotransfer of Cry1C protein expressed in T1C-19 rice to *N. lugens*, and its subsequent transfer to *P. fuscipes* was investigated. To further verify whether Cry1C protein had any direct toxicity to *P. fuscipes*, a Tier-1 bioassay was performed in which *P. fuscipes* adults were fed an artificial diet incorporating purified Cry1C protein at a level much higher than that likely to be encountered under field conditions. Also, this study was to explore whether Cry1C protein could be transferred to *P. fuscipes* through BPH by Enzyme-linked immunosorbent assay (ELISA).

## Results

### Effects of prey sources on the survival and development of *P. fuscipes*

The whole preimaginal development of *P. fuscipes* fed *N. lugens* nymphs was 28.9 d (Table 1). When incorporated with *Drosophila melanogaster* adults, the first instar, second instar, pupa and whole preimaginal durations were significantly shortened (P < 0.05) (Table 1). However, the preimaginal survival rate, sex ratio and female and male weight were not significantly affected by prey source (P > 0.05) (Table 1). The results showed that the food combination containing *N. lugens* nymphs added with *D. melanogaster* adults could shorten preimaginal stage duration of *P. fuscipes*.

### Impact of different artificial feeding system on development of *P. fuscipes*

Compared to *P. fuscipes* larvae fed the artificial diet only, the larval survival rate increased from 52.0 to 68.0% (P = 0.046) when their artificial diet incorporated nymphs of *N. lugens* (Table 2). The first and second instar larvae, pre-pupa and whole preimaginal durations were significantly shortened (P < 0.05), and the female body weight increased significantly (P < 0.05) (Table 2). When not provided with the artificial diet, a group of 45 *P. fuscipes* larvae all died within 6 d, indicating that they could not survive by feeding only on humus of soil. The results showed that if *P. fuscipes* larvae developed into adults, they must have consumed the artificial diet, which could then be used as a medium to deliver Bt protein into *P. fuscipes*.

### Life-table parameters of *P. fuscipes* fed on transgenic *cry1C* rice

There were no significant differences in preimaginal developmental time, preimaginal survival, female ratio, pre-oviposition, total fecundity and fresh body weight between *P. fuscipes* reared with first to third instar nymphs of brown planthopper fed on transgenic *cry1C* rice compared with non-Bt rice (P > 0.05) (Table 3).

### Cry1C protein detection in rice plants, larvae of *N. lugens* and *P. fuscipes*

**Figure 1:**
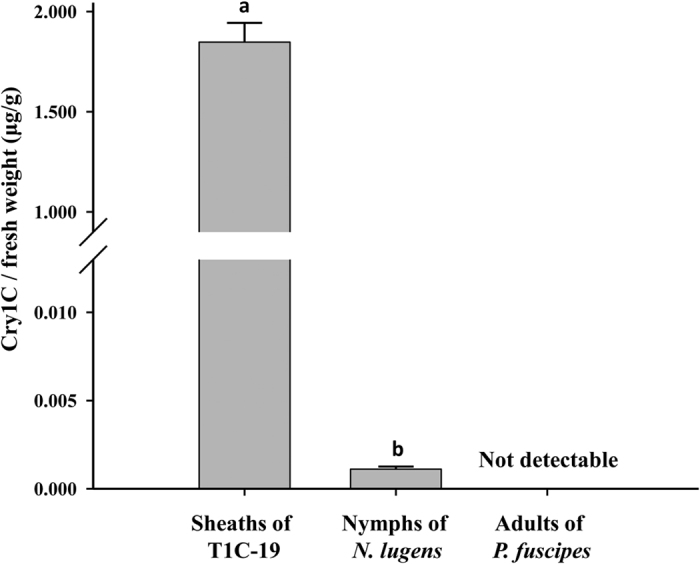
Concentrations (Mean ± SE) of Cry1C in T1C-19 rice sheath, nymphs of *N. lugens* and larvae of *P. fuscipes*.

ELISA assay showed that the concentration of Cry1C protein in rice sheaths was 1.8 ± 0.09 μg/g fresh weight. When first to third instar nymphs of *N. lugens* were reared on Bt rice, the Cry1C protein concentration in nymphs was 1.1 ± 0.0 ng/g. When *P. fuscipes* larvae preyed on first to third instar *N. lugens* nymphs fed on T1C-19, no Cry1C protein was detected in newly emerged *P. fuscipes* adults (Fig. 1).

### Purified Cry1C protein bioassay and life-table parameters of *P. fuscipes*

The Cry1C concentrations in the artificial diets were 12.4 ± 0.9 and 11.5 ± 0.1 μg/g, respectively, before and after exposure to *P. fuscipes* for 24 h. No significant differences were observed in the Cry1C concentration in diets (the Cry1C concentration decreased by 7.2%) before and after exposure (Student’s t-test, P = 0.422). This indicated that Cry1C concentration was stable in the artificial diets throughout the feeding process.

The LC50 (concentration resulting in 50% mortality) of this batch of Cry1C for *Plodia interpunctella* larvae was 0.03 μg/g fresh weight. Before and after exposure to *P. fuscipes* for 24 h, the mortalities of *C. medinalis* larvae were 55.6% and 48.9%, respectively, fed on the artificial diets with 50 μg/g Cry1C potein. However, the mortality of *C. medinalis* larvae was 17.8% after feeding on the pure artificial diet. The results suggested that the artificial diets with Cry1C protein had insecticidal activity for *P. fuscipes*.

**Figure 2:**
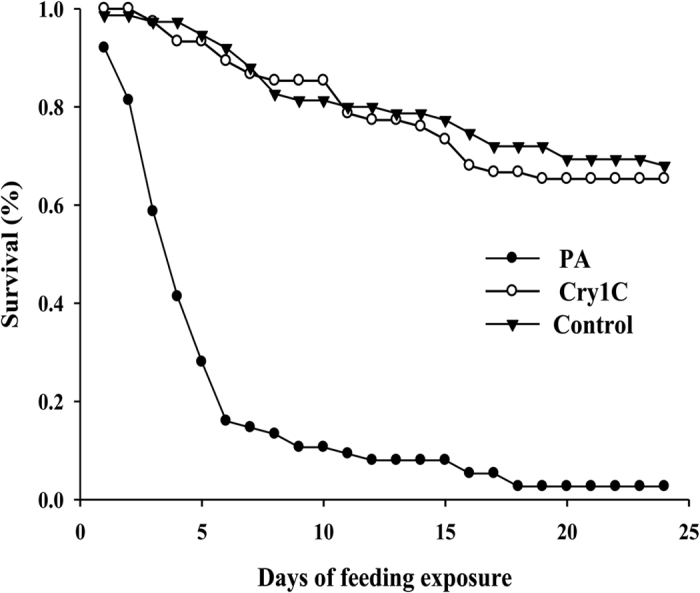
Survival of larvae of *P. fuscipes* feeding on artificial diet or artificial diet containing Cry1C/PA (50 μg/g Cry1C or 1 mg/g PA) (N = 75).

Significantly lower survival rate was observed in *P. fuscipes* fed on artificial diet containing 1 mg/g potassium arsenate (PA) relative to the pure artificial diet (P < 0.001, Fig. 2). No significant difference (P = 0.729, χ^2^ = 0.1210, d.f. = 1) was detected in the larval survival rate of *P. fuscipes* fed an artificial diet containing 50 μg/g Cry1C protein compared with pure artificial diet (Table 4). Similarly, no differences were found in the developmental duration, preimaginal survival rate and female ratio of *P. fuscipes* with high dosage of Cry1C compared to the pure artificial diet (Table 4).

## Discussion

Rove beetle (*P. fuscipes*) is widely distributed in all temperate and tropical continents^23^. It lives in humid habitats such as marshes, edges of freshwater lakes and streams, and rice fields^14^,^24^. It preys on soft-bodied insect pests such as aphids, whitefly, mites, maggots of fruit fly and leaf hoppers of different crops^25^. Both the larvae and adults of *P. fuscipes* forage for food on the foliage or among tillers of rice plants^26^. They are important predators in rice fields and effective biological control agents against *N. lugens*, which is the most important pest in tropical rice fields and the main non-target insect pest in Bt rice fields^27^. Bt protein could expose the predator *P. fuscipes* insects through predation of the non-target herbivores *N. lugens*. Therefore, *P. fuscipes* is a good surrogate for non-target arthropods (NTAs), and it is vital to evaluate the effects of Bt rice on *P. fuscipes* prior to any commercialization. There are few reports on safety evaluation of Bt crops on *P. fuscipes*. There was no impact on the survival and predation function of adult *P. fuscipes* fed *N. lugens* reared with transgenic *cry1Ac/cry1Ab* rice in laboratory studies^28^; and the Cry1Ab protein expressed by maize hybrid MON810 did not influence the overall community structure of the rove beetle in a field experiment^29^.

Romeis *et al*.^10^ and Yu *et al*.^30^ descried the ecological risk assessment of transgenic plant for NTAs. In the present study, the ecological risk of transgenic *cry1C* rice to *P. fuscipes* was developed by two experiments: (1) a direct feeding experiment, in which *P. fuscipes* was fed an artificial diet containing Cry1C at a dose 10 times that it may encounter in realistic field conditions; and (2) a tritrophic experiment, in which the Cry1C protein was delivered to *P. fuscipes* indirectly through preying on *N. lugens* nymphs. The transgenic *cry1C* rice and Cry1C protein had no significant detrimental effects on the developmental time, preimaginal survival, female ratio and body weight of *P. fuscipes*. This is the first report of an assessment continuum for the effects of transgenic *cry1C* rice on *P. fuscipes*.

“Tier-1 assays” is the initial step to determine the potential hazard or toxicity of the insecticidal compound produced by GE plants, such as a Cry protein, to selected test species^31^. In the present study, a direct feeding experiment was carried that purified Cry1C protein was fed to *P. fuscipes* larvae by reference to the method of Zhou^32^. We selected artificial diets combining nymphs of *N. lugens* to deliver Cry1C protein into *P. fuscipes*. The oral poison potassium arsenate (PA), as a positive control, was used to verify the dietary exposure assay. This experiment showed that significantly lower survival was found in *P. fuscipes* fed on artificial diet containing 1 mg/ml PA relative to those fed on pure artificial diet, which indicated the test system in this experiment could be used to detect the dietary effects of insecticidal compounds potassium arsenate. No negative effect was found in the life-table parameters of *P. fuscipes* when provided with the artificial diet containing Cry1C at a concentration 10 times that for actual exposure concentration in the field compared with the pure artificial diet (negative control). Our experiment was the first report to evaluate the potential toxicity effect of Cry1C on *P. fuscipes* by use of “Tier-1 assays”.

The present study showed that different prey sources affected development of *P. fuscipes*. The experiment started with 50 *P. fuscipes* larvae when fed on *N. lugens*. To ensure enough prey samples, 80 *P. fuscipes* larvae fed on *N. lugens* added *D. melanogaster* as a supplement. The results showed that feeding *P. fuscipes* with both *N. lugens* nymphs and *D. melanogaster* adults significantly shortened growth duration, compared to feeding *P. fuscipes* with only *N. lugens* nymphs. This is consistent with Bong’s findings that survival and adult fecundity of *P. fuscipes* were negatively affected when lobster cockroach *Nauphoeta cinerea* (Olivier) was used as the only food resource^33^. Many reports have also shown that different nutritional composition of prey may alter biological parameters of predators^34^,^35^,^36^,^37^.

Brown planthopper is the important non-target herbivore in transgenic Bt rice. It piercing and sucking phloem of rice. Bt proteins of transgenic rice could be transferred to it via feeding^38^. However, whether Bt protein could be transferred to predators of brown planthopper are controversial. Cry1Ab and Cry2A could be transferred to *Cyrtorhinus lividipennis* and *Hylyphantes graminicola* by predation on brown planthopper fed on transgenic Bt rice^39^,^40^. While Cry2A protein could not be transferred to *C. sinica* and *C. lividipennis* via preying *N. lugens* fed on transgenic Bt rice^38^. In the current study, Cry1C also could not be transferred to *P. fuscipes* via predation of *N. lugens*. Even different predators preying *N. lugens* fed on the same transgenic Bt rice containing different concentration of Bt protein in the body. Further study should be focused on whether this difference is caused by the different feeding behavior of predator.

Our study was the first to elaborate the effects of transgenic *cry1C* rice on *P. fuscipes*, a predator in rice ecosystems, and utilized a Tier-1 examination system and tritrophic bioassay. The results indicated that *P. fuscipes* was not sensitive to Cry1C protein and transgenic *cry1C* rice (T1C-19) poses a negligible risk to *N. lugens*.

## Materials and Methods

### Plant materials

The transgenic rice line, T1C-19 and its corresponding non-transformed parental rice Minghui 63 were used for the experiments. T1C-19 expresses a gene encoding synthetic *cry1C* under the control of the corn ubiquitin promoter and exhibits high resistance to stem borers and leaffolders^16^. Both rice lines were obtained from National Key Laboratory of Crop Genetic Improvement, Huazhong Agricultural University (Wuhan, China). The culture methods of both rice lines for the laboratory experiments were described by Han *et al*.^38^ and Yoshida *et al*.^41^.

### *N. lugens* and *P. fuscipes*

The original adults of *N. lugens* were randomly collected from paddy fields in Wuhan, Hubei Province, China. Prior to the tritrophic bioassay, *N. lugens* were reared on T1C-19 and Minghui 63, respectively, for more than ten generations to obtain uniform colonies. *N. lugens* was fed on 15-day-old rice seedlings cultured with Yoshida solution in plastic tanks.

The original individuals of *P. fuscipes* were also randomly collected from paddy fields in Xiaogan city. Both artificial diets and *N. lugens* nymphs were supplied to *P. fuscipes* larvae as their food source and maintained in the laboratory for more than one generation. The larvae were reared in plastic containers (22 cm length × 7 cm width × 7 cm height) covered with nylon mesh on the open end. First, a piece of wetted sponge was placed at the bottom of the container to maintain humidity, then the container was filled up to 2–2.5 cm with soil (damp soil rich in humus for raising maize) to provide a natural environment. There were 30–50 rice seedlings put into the container to feed *N. lugens* nymphs (100–300 first–third instar) and the roots of rice seedlings were supplied with moist cotton to maintain moisture. Meanwhile, artificial diets were put on a glass slide that was placed on the soil. In each container, 20–50 larvae of *P. fuscipes* were reared. The artificial diet was refreshed daily, and at least 100 nymphs of *N. lugens* were supplied into each container daily. The artificial diet was prepared as described by Zhou^32^ with some modification. Liver powder purchased from Nutritional Food Co. Ltd., Yi Wei, China, Product Code: 1047457832 (specification: 3.5 g × 15 package; nutrient content: pork liver 50%, chicken liver 20%, goose liver 15%, jujube 10% and galacto-oligosaccharides 5%) and honey (acacia honey from Kangsi farmers, net 450 g, Huazhong Agricultural University) were mixed at the ratio of 5:1, and stirred with a glass rod for 1 min or more.

Newly-eclosed adults were picked out with a brush and reared in a glass beaker. The *P. fuscipes* adults were supplied with both *N. lugens* nymphs and with artificial diets. Into a 100-mL glass beaker, 200 rice seedlings were placed and reared with Yoshida culture solution, and 200–400 *N. lugens* nymphs were raised on the rice seedlings. Then, this glass beaker was placed inside a 2000-mL glass beaker (with a piece of wetted sponge at the bottom to maintain humidity), as also was a glass Petri dish (5 cm diameter × 5 cm height) holding the artificial diet. The females laid eggs on sponge and rice seedlings. After 5 days of oviposition, the sponge and rice seedlings were transferred to a plastic container (22 cm length × 7 cm width × 7 cm height), with wetted tampons to maintain humidity. When larvae hatched, they were transferred to a plastic container for larvae rearing as described previously. All the insects were cultured and all experiments were conducted in a climatic chamber at 28 ± 1 °C, relative humidity 70 ± 5% and a light/dark cycle of 14/10 h.

### Effects of prey sources on survival and development of *P. fuscipes*

Because *P. fuscipes* is a polyphagous predator in rice fields, before we conducted the prey-mediated effects of transgenic *cry1C* rice on *P. fuscipes*, it was essential to evaluate the effects of a single prey source on survival and development of *P. fuscipes*. If the single prey source had negative impacts on *P. fuscipes*, then another prey source should be added to minimize the negative effects. Therefore, two kinds of prey were supplied to *P. fuscipes*. Newly hatched larvae of *P. fuscipes* (<24 h) were put individually into glass tubes (2 cm diameter × 12 cm length) sheathe with nylon mesh. In order to maintain humidity, each glass tube was filled with a piece of wetted sponge in the bottom: (i) 9–10 nymphs of *N. lugens* (first–third instar), reared on TN1 rice plants, were used daily as the prey of *P. fuscipes*; (ii) in addition to 9–10 *N. lugens* nymphs daily, two adults of *D. melanogaster* (supplying one adult of *D. melanogaster* at 2 d after *P. fuscipes* neonates hatched, 2d at second-instar larvae of *P. fuscipes*, respectively) were also employed as prey of *P*. *fuscipes*. The survival and ecdysis of insects were recorded daily. Sex and body weight of rove beetle were recorded after *P. fuscipes* adults were emerged.

### Effects of different artificial rearing system on the survival and development of *P. fuscipes*

Newly hatched larvae (<48 h) of *P. fuscipes* were individually reared in Petri dishes (5 cm diameter × 5 cm height) to evaluate whether the artificial diets met the growth and development demands of *P. fuscipes*, and whether the *P. fuscipes* larvae could survive by only feeding on humus in soil. Each Petri dish contained damp soil rich in humus (1 cm in thickness) at the bottom, and three different artificial rearing systems were constructed: (i) damp soil only; (ii) damp soil and artificial diet; and (iii) damp soil and artificial diet plus nymphs of *N. lugens* (supplying 10 nymphs of *N. lugens* at 6, 7 and 8 d after *P. fuscipes* feeding artificial diets, respectively). The survival and ecdysis of insects were recorded daily. Sex and body weight of rove beetle were recorded after *P. fuscipes* adults were emerged.

### Effects of Bt-rice on the life-table parameters of *P. fuscipes* fed on *N. lugens* nymphs

Newly molted first instar larvae (<24 h) of *P. fuscipes* were put individually into glass tubes (2 cm diameter × 12 cm height) covered with nylon mesh. In order to maintain humidity, each glass tube was filled with a piece of wetted sponge in the bottom. One 15-d-old rice seedling was introduced into the glass tube as food for 9–10 (first–third instar) nymphs of *N. lugens*. Therefore, *P. fuscipes* larvae could prey on *N. lugens* nymphs. The rice seedlings and nymphs of *N. lugens* were changed daily. Two adults of *D. melanogaster* (supplying one adult of *D. melanogaster* at 2 d after *P. fuscipes* neonates hatched, 2 d at second-instar larvae of *P. fuscipes* respectively) were also employed as prey of *P. fuscipes*. The survival and ecdysis of *P. fuscipes* nymphs were monitored every day. The sex and body weight of *P. fuscipes* adults were recorded upon emergence of adults. For each rice line, 80 larvae of *P. fuscipes* were tested. Upon early emergence of the *P. fuscipes,* male and female were paired and reared in in a plastic petri dish (9cm in diameter) that contained a moist filter paper, and was supplied 15 adults of *N. lugens* fed on transgenic cry1C rice daily. Moist cotton was introduced into petri dish and provided as the water supply and oviposition site. Then every dish was covered with parafilm sealed edge and small hole left unsealed for breathing. Egg number of each pair was recorded daily. Ten pairs were tested for each rice line. One moist cotton was put into each well of 24-well tissue culture plate for maintaining humidity. Eggs were collected with a brush, and were put individually into each well. Egg hatching was observed every day and recorded. Thirty eggs for one replicate, and three replicates for each rice line.

### Cry1C detection in rice plants, *N. lugens* and *P. fuscipes*

The sheaths of 15-d-old rice seedlings, nymphs of brown planthopper (first to third instar) fed on T1C-19 or Minghui 63, and newly emerged adults of *P. fuscipes* that preyed on first to third instar *N. lugens* nymphs were collected for detection of the Cry protein. For each treatment, five samples of rice sheaths (20 mg per sample), three samples of *N. lugens* (approximate 35 mg per sample) and three samples of *P. fuscipes* adults (approximate 35 mg per sample) were tested. The methods of Cry protein contents were determined by Han *et al*.^38^.

### Exposure of *P. fuscipes* to high dose of Cry1C

Lyophilized Cry1C protein was purchased from the Biochemistry Department Laboratory, School of Medicine, Case Western Reserve University, USA. The methods of purifying and lyophilizing Cry protein were described by Han *et al*.^38^.

*P. fuscipes* were reared on the artificial diet containing Cry1C protein. Three different dietary treatments were delivered to the first instar nymphs of *P. fuscipes*: (i) A pure artificial diet (negative control) and 30 (first–third instar) nymphs of *N. lugens* (respectively supplying 10 nymphs of *N. lugens* at 6, 7 and 8 d after *P. fuscipes* were fed artificial diets); (ii) An artificial diet containing 50 μg/g of Cry1C and 30 nymphs (first to third instar) of *N. lugens* (respectively supplying 10 nymphs of *N. lugens* at 6, 7 and 8 d after *P. fuscipes* were fed artificial diets); (iii) An artificial diet containing 1 mg/g of potassium arsenate (PA, positive control) and 30 nymphs (first to third instar) of *N. lugens* (respectively supplying 10 nymphs of *N. lugens* at 6, 7 and 8 d after *P. fuscipes* were fed). Three different diets were refreshed daily. Simultaneously, survival and molting of *P. fuscipes* were recorded daily. Sex and body weights of *P. fuscipes* were recorded when the adults were emerged. For each treatment, 75 *P. fuscipes* larvae were evaluated.

The artificial diet mixed with Cry1C was fed to *P. interpunctella* to determine Cry1C bioactivity on lepidopteran insects. Each bioassay included six concentrations of Cry1C (0, 0.02, 0.05, 0.08, 0.11 and 0.14 μg/g). For each concentration there were 40 newly hatched *P. interpunctella* larvae introduced on the artificial diets and 5 replicates were tested. After one week, the mortality of the larvae was recorded. The LC50 of this batch of Cry1C protein was measured.

In order to ensure the stability and the bioactivity of Cry1C protein of artificial diets that taken from the freezer and from diets that had been exposed to *C. medinalis* for 24 h, the Cry1C proteins were extracted from artificial diets and the methods described by Han *et al*.^38^. After 2 h of air-drying, fifteen Bt-susceptible first-instar *C. medinalis* were used in each treatment. And each treatment was three replicates. Mortality of the insects was calculated after 48 hours.

### Data analysis

In all bioassays with *P. fuscipes*, statistical comparisons were made between each treatment and the control (pure diet). Student’s t-tests were used to compare the data of body weight. Mann–Whitney U-test was used to analyze the developmental time of nymphal. The parameters of preimaginal survival and female ratio were used by the Chi-square test. And the percentage data were arcsine–square root and transformed by SQRT (χ + 1) or log 10 (χ + 1). The effect of Cry1C protein dietary treatments on *P. fuscipes* survival was analyzed with the Kaplan-Meier procedure and log-rank test. All statistical analyses were conducted by the software package SPSS (version 16.0 for Windows, 2007).

## Additional Information

**How to cite this article**: Meng, J. *et al*. No impact of transgenic *cry1C* rice on the rove beetle *Paederus fuscipes*, a generalist predator of brown planthopper *Nilaparvata lugens*. *Sci. Rep.* **6**, 30303; doi: 10.1038/srep30303 (2016).

## Figures and Tables

**Table 1:** Impact of prey sources on the survival and development of *P. fuscipes*.

Parameter	Food combination		Statistics
	*N. lugens*	*N. lugens* with Drosophila melanogaster	
1^st^ instar developmental time (days ± SE)^a^	6.2 ± 0.17 (45)^a^	4.4 ± 0.11 (65)^b^	U = 376.5, P = 0.000
2^nd^ instar developmental time(days ± SE)^a^	16.9 ± 0.91 (33)^a^	8.6 ± 0.49 (47)^b^	U = 43.000, P = 0.000
Pre-pupa developmental time (days ± SE)^a^	1.7 ± 0.14 (21)^a^	1.7 ± 0.10 (35)^a^	U = 356.500, P = 0.834
Pupa developmental time (days ± SE)^a^	4.1 ± 0.24 (19)^a^	3.5 ± 0.10 (32)^b^	U = 177.000, P = 0.007
Preimaginal stage duration (days ± SE)^a^	28.9 ± 1.04 (19)^a^	17.7 ± 0.41 (32)^b^	U = 0.000, P = 0.000
Preimaginal survival (%)^b^	38.0^a^	40.0^a^	χ2 = 0.052, P = 0.820
Female ratio (%)^b^	52.6^a^	56.2^a^	χ2 = 0.063, P = 0.802
Female body weight (mg ± SE)^c^	2.88 ± 0.15^a^	3.08 ± 0.08^a^	t = −1.284, P = 0.211, d.f = 26
Male body weight (mg ± SE)^c^	2.61 ± 0.12^a^	2.86 ± 0.11^a^	t = −1.518, P = 0.144, d.f = 21

(1) The experiment started with 50 larvae when fed on *N. lugens*, and 80 larvae fed on *N. lugens* added D. melanogaster as a supplement, (2) number of replicates is given in parentheses per treatment, (3) data (means ±  SE) in a line followed by the same letter are not significantly different (P > 0.05), (4).

^a^Mann-Whitney U-test.

^b^Chi-square test.

^c^student’s t-test.

**Table 2:** Impacts ofdifferent artificial feeding systems on the survival and development of *P. fuscipes*.

Parameter	Treatments		Statistics
	Artificial diet	Artificial diet + *N. lugens*	
1^st^ instar developmental time (days ± SE)^a^	6.9 ± 0.37 (63)^a^	4.4 ± 0.24 (65)^b^	U = 943.5, P = 0.000
2^nd^ instar developmental time(days ± SE)^a^	13.0 ± 0.49 (44)^a^	8.4 ± 0.28 (59)^b^	U = 53.0, P = 0.000
Pre-pupa developmental time (days ± SE)^a^	1.7 ± 0.11 (42)^a^	1.4 ± 0.07 (55)^b^	U = 865.0, P = 0.016
Pupa developmental time (days ± SE)^a^	3.2 ± 0.12 (39)^a^	3.4 ± 0.08 (51)^a^	U = 919.5, P = 0.486
Preimaginal stage duration (days ± SE)^a^	24.6 ± 0.66 (39)^a^	17.2 ± 0.31 (51)^b^	U = 39.0, P = 0.000
Preimaginal survival (%)^b^	52.0^a^	68.0^b^	χ2 = 0.046, P = 0.046
Female ratio (%)^b^	35.9^a^	51.0^a^	χ2 = 1.396, P = 0.237
Female body weight (mg ± SE)^c^	2.79 ± 0.13^a^	3.14 ± 0.08^b^	t = −2.41, P = 0.021, d.f = 39.0
Male body weight (mg ± SE)^c^	2.78 ± 0.08	2.60 ± 0.05	t = 1.994, P = 0.053, d.f = 38.7

(1) The experiment started with 50 larvae when fed on *N. lugens*, and 80 larvae fed on *N. lugens* added D. melanogaster as a supplement, (2) number of replicates is given in parentheses per treatment, (3) data (means ± SE) in a line followed by the same letter are not significantly different (P > 0.05), (4).

^a^Mann-Whitney U-test.

^b^Chi-square test.

^c^student’s t-test.

**Table 3:** Prey-mediated effects of Cry1C on the life-table parameters of *P. fuscipes* preying on *N. lugens* nymphs reared on T1C-19 and Minghui 63 rice plants.

Parameter	Rice Varieties		Statistics
	T1C-19	Minghui 63	
1st instar developmental time (days ± SE)^a^	4.4 ± 0.15 (73)	4.4 ± 0.11 (76)	U = 2.680, P = 0.707
2nd instar developmental time (days ± SE)^a^	8.4 ± 0.42 (54)	8.6 ± 0.49 (47)	U = 10.000, P = 0.326
Pre-pupa developmental time (days ± SE)^a^	1.6 ± 0.09 (36)	1.7 ± 0.10 (35)	U = 558.500, P = 0.349
Pupa developmental time (days ± SE)^a^	3.7 ± 0.12 (33)	3.5 ± 0.10 (32)	U = 436.000, P = 0.177
Preimaginal stage duration (days ± SE)^a^	16.7 ± 0.39 (33)	17.7 ± 0.41 (32)	U = 384.000, P = 0.056
Preimaginal survival (%)^b^	41.3	40.0	χ2 = 0.026, P = 0.872
Female ratio (%)^b^	48.5	56.2	χ2 = 0. 393, P = 0.531
Female body weight (mg ± SE)^c^	3.07 ± 0.15	3.08 ± 0.08	t = 0.054, P = 0.957, d.f = 32
Male body weight (mg ± SE)^c^	2.78 ± 0.10	2.86 ± 0.11	t = 0.556, P = 0. 583, d.f = 29
pre-oviposition (days ± SE)^a^	21.0 ± 3.54 (10)	11.8 ± 1.69 (10)	U = 25.500, P = 0.111
Fecundity (n ± SE)^c^	97.20 ± 17.86 (10)	96.0 ± 12.55 (10)	t = −0.055, P = 0.957
Egg hatchability (%)^c^	0.92 ± 0.04	0.90 ± 0.02	t = −0.667, P = 0.543, d.f = 4

(1) The experiment started with 80 larvae per treatment, (2) number of replicates is given in parentheses per treatment, (3) data (means ± SE) in a line followed by the same letter are not significantly different (P > 0.05), (4).

^a^Mann-Whitney U-test.

^b^Chi-square test; student’s t-test.

**Table 4:** Effect of high dosage of purified Cry1C incorporated into the artificial diet on the life-table parameters of *P. fuscipes*.

Parameter	Treatments		
	Artificial diet	Artificial diet + 50 μg/g *Cry1C*	Artificial diet + 1 mg/g PA
1^st^ instar developmental time (days ± SE)^a^	4.4 ± 0.24 (65)	5.0 ± 0.35 (62)	3.9 ± 0.76
2^nd^ instar developmental time (days ± SE)^a^	8.4 ± 0.28 (59)	8.2 ± 0.32 (52)	—
Pre-pupa developmental time (days ± SE)^a^	1.4 ± 0.07 (55)	1.3 ± 0.07 (50)	—
Pupa developmental time (days ± SE)^a^	3.4 ± 0.08 (51)	3.3 ± 0.07 (49)	—
Preimaginal stage duration (days ± SE)^a^	17.2 ± 0.31 (51)	17.4 ± 0.48 (49)	—
Preimaginal survival (%)^b^	68.0	65.3	0.027^**^
Female ratio (%)^b^	51.0	38.8	—
Female body weight (mg ± SE)^c^	3.1 ± 0.08	2.9 ± 0.07	—
Male body weight (mg ± SE)^c^	2.6 ± 0.05	2.7 ± 0.07	—

The experiment started with 75 larvae per treatment; the experiment lasted until adults eclosed. Larvae of *P. fuscipes* were fed with an artificial diet containing 50 μg/g Cry1C or 1 mg/g PA (positive control). Pure diet served as a negative control. Number of individuals at each development stage is given in parentheses. Statistical comparisons were made separately for each of the insecticidal compounds compared with the control. Asterisks denote significant differences: P < 0.01.

^a^Mann–Whitney U-test.

^b^Chi-square test.

^c^Student’s t-test.
